# New Findings in *Prunus padus* L. Fruits as a Source of Natural Compounds: Characterization of Metabolite Profiles and Preliminary Evaluation of Antioxidant Activity

**DOI:** 10.3390/molecules23040725

**Published:** 2018-03-22

**Authors:** Dario Donno, Maria Gabriella Mellano, Marta De Biaggi, Isidoro Riondato, Ernest Naivonirina Rakotoniaina, Gabriele Loris Beccaro

**Affiliations:** 1 Dipartimento di Scienze Agrarie, Forestali e Alimentari, Università degli Studi di Torino, Largo Braccini 2, 10095 Grugliasco, Italy; gabriella.mellano@unito.it (M.G.M.); marta.debiaggi@unito.it (M.D.B.); isidoro.riondato@unito.it (I.R.); gabriele.beccaro@unito.it (G.L.B.); 2 Département de Biologie et Écologie Végétales, Faculté des Sciences, Université d’Antananarivo, 101 Antananarivo, Madagascar; rakotoniainae@yahoo.fr; 3 IMRA—Institute Malgaches des Recherches Appliqués, 101 Antananarivo, Madagascar

**Keywords:** bird cherry, fruit healthy properties, biodiversity, phytochemicals, traditional food, chromatographic fingerprinting, antioxidants

## Abstract

European bird cherry (*Prunus padus* L.) has been known since the Middle Ages for its medical/food use and high health-promoting value. This study aimed to assess the potential of these fruits as a source of bioactive compounds through the characterization of its physicochemical traits, nutraceutical properties, phytochemical composition via HPLC fingerprint, and antioxidant capacity. Fully ripened fruits of *Prunus padus* L. (Colorata cv) were collected in mid-July 2017 in Chieri, north-western Italy. The TPC (194.22 ± 32.83 mg_GAE_/100 g_FW_) and TAC (147.42 ± 0.58 mg_C3G_/100 g_FW_) values were obtained from the analyzed extracts. The most important phytochemical class was organic acids (48.62 ± 2.31%), followed by polyphenols (35.34 ± 1.80%), monoterpenes (9.36 ± 0.64%), and vitamin C (6.68 ± 0.22%). In this research the most important flavonols selected as marker were quercitrin (16.37 ± 3.51 mg/100 g_FW_) and quercetin (11.86 ± 2.36 mg/100 g_FW_). Data were reported based on fresh weight. Moreover, fresh fruits showed a mean antioxidant activity value of 17.78 ± 0.84 mmol Fe^2+^·kg^−1^. Even though the seeds and leaves contain cyanogenic glycosides, this study showed that these fruits could be a natural source of bioactive compounds with high antioxidant properties, due to the contents of organic and phenolic acids, catechins, and a synergetic effect of vitamin C and flavonoids.

## 1. Introduction

In recent years, a global interest in the traditional use of plants in folk medicinal and food applications was observed, also in many European countries. Unfortunately, information on traditional use of these neglected fruits is scarce, but it is very important for the evaluation of the chemical composition and biological activity (antioxidant capacity) of fruits used in traditional medical and food systems. The correlation between the use of traditional food resources and culinary practices with people’s health is also of great importance. Much of the knowledge has been developed before conventional medications were introduced; however, some studies reported the remaining recipes of traditional remedies based on neglected fruits [1].

European bird cherry (*Prunus padus* L.) is a boreal-temperate Eurasian species, known since the Middle Ages for its medical/food use and high health-promoting value. The species, which belongs to the *Prunus*-genus of *Rosaceae* family, is widely distributed in Europe, in woods or river banks. Wild *Prunus*-species belong to three subgenera: *Prunus* (plum-species), *Cerasus* (cherry-species), and *Padus* (bird cherry-species). It grows quickly and has strong adaptability [1]. Bird cherry growth power is related to the latitude of growing-site, habitats and age: growth is fastest and most variable at young age and on rich loamy soils overlying limestone [2]. There are three bird cherry subspecies (*padus*, *borealis*, *petraea*) depending on the differences in morphology and distribution area.

*Prunus padus* belongs to the same genus of several cultivated species as almond, peach and nectarine, plum, sloe and sweet cherry, and it has also been used as graftstock for sour cherry: it can be potentially used as a gene donor for crop improvement. It is an excellent garden ornamental plant: in the past this species was often moved from natural growing-sites into home gardens especially in the areas where the climate is harsh (northern Europe). Bird cherries are easy to cultivate for many reasons: they grow fast and are easily reproduced by seeds or by bending twigs which root easily. Bird cherries can also produce ground shoots and root suckers [3].

European bird cherries are host plants for many herbivores and pathogens and for this reason they were sometimes removed from urban or rural sites. The subspecies *borealis* is rare and locally threatened in some countries as identified by International Union for Conservation of Nature (IUCN) in the “Red List of Threatened Species” European assessment: therefore, a national level monitoring is required. Germplasm collection, incorporation of targeted monitoring, management of this species in protected areas where it is present, and duplicated ex situ storage are priorities for this species.

This deciduous tree or shrub presents ornamental flowers and edible fruits [4]. *P. padus* is a honey plant and germplasm resource because the flower has a good nectar [1]. It flourishes in May and June and its stone fruits with dark purple-red skin and light pulp have high ornamental value. The sticky and flavorless fruits ripen from July to September and fall early [5]. Bird cherry is a subglobose or globose-ovoidal drupe, 10–12 mm long and about 10 mm wide with a weight of 2–3 g. Bird cherries seem to produce very active metabolites, as cyanogenic glycosides and specific phenols, for qualitative defenses of valuable immature vegetative parts and seeds against herbivores [4]. Fruits contain up to 5% of sugars, oxalic and citric acids, a lot of tannins, terpenes, and volatile oils. Most of the phytochemical compounds (flavonoids, anthocyanins, phenolic acids, as well as vitamins) present in *P. padus* fruits, exhibit a proved and high antioxidant capacity [6]: these molecules inhibit the free radical generation and prevent protein, lipids, and also nucleic acids oxidative damage [7].

Bird cherry shows a high chemical variability: flowers, fruits, and leaves are used for relieving cough and invigorating the spleen. People have utilized the bark as pesticide before the industrial products were available [4]. For example, the bark was spread on potato fields and apple orchards for its smell and bitter taste. For several years its cyanogenic glycosides and phenols protected cultivations from rodent and insect attacks. Fruits, bark, powdered leaves, and flowers are still used as anesthetics and disinfectants in Russia and in Scandinavian countries. Moreover, fruits can solve dysentery and digestion problems and stomach-ache (similar to blueberries). People in Eastern and Northern Europe usually dry, crush, and grain the fruits, use them in tart fillings or mix them up with flour for baking. Russians make bird cherry liqueur or thickened juices. The fruits are added to alcoholic beverages or juices and dye the liquids dark red [4].

Recently, traditional food plants have become very attractive to the food industry, prompting their use as replacements for synthetic chemicals and nutraceuticals, but neglected and underutilized natural food resources, as *P. padus*, are suffering from few attention and research, and their phytochemical potential is not fully exploited. Therefore, investigation of such properties has been of interest mainly for finding new sources for natural antioxidants for food supplements or other food applications: in this sense, European bird cherry is a very important resource for its phytochemical composition, nutritional value, and antioxidant properties. This work aimed at describing bird cherry fruit physicochemical traits and evaluate its potential as a source of natural bioactive compounds through the characterization of its nutraceutical properties and antioxidant capacity. Furthermore, the assessment and quantification of several phytochemicals, selected as health-promoting markers with demonstrated efficacy on the human organism, were performed using high-performance liquid chromatography-diode array detection (HPLC-DAD) to define plant material as potential health-promoting foods.

## 2. Results and Discussion

### 2.1. Morphological and Quality Parameters

Table 1 presents the fruit weight and size, total soluble solids (TSS), pH, and titratable acidity (TA) of the analyzed bird cherry genotype. Results showed that the bird cherry fruit is quite spheroidal in shape (11.11 ± 0.08 mm in length and 11.94 ± 0.09 mm in width), with a mean weight value of 2.08 ± 0.02 g and a dark red color.

Quality analysis produced a mean TSS value of 16.17 ± 0.45 Brix, while TA showed a mean value of 245.33 ± 13.32 meq·L^−1^ with a pH mean value of 2.93 ± 0.03 in accordance with previous studies [4]. In the present research, ripe bird cherry fruits contained significantly high levels of TSS. However, sugar contents might be significantly affected by the process of maturation: indeed, individual and total sugars content increase with maturity due to starch mobilization and translocation from other plant organs as reported by Mikulic-Petkovsek et al. [8] for different fruit species. Moreover, TA and relative pH value determine consumer perceptions of fruit sweetness and sourness. Titratable acidity significantly decreases at full maturity and the lowest levels are generally measured at the last harvest stage: in this study, only one maturation stage (ripe fruits for fresh consumption) were considered in order to better characterize the *P. padus* fruit quality as health-promoting fresh product. Sugar to acid ratio represents the ratio between total sugars (TSS) and total organic acids (TA) content in fruits: the sugar/acid ratio, important for the taste and flavor of fruits, is often used as an index of sweetness for specific fruits. In this research, bird cherry fruits showed a high sugar to acid ratio value (9.80), due both to high TSS value and to low TA value.

### 2.2. Nutraceutical Properties

Polyphenols are molecules with demonstrated positive effects against oxidative stress and inhibition of macromolecular oxidation. Moreover, a considerable interest in the potential health-promoting and protective effects of anthocyanin-rich fruits or their derived-products in humans and animals has been recently reported [9]. These molecules are mainly found in the external layers of the skin while flesh tissue contains little or no anthocyanins [10] as in *Prunus padus* fruits. The evolution of anthocyanins is parallel to sugar accumulation, but no direct relationship has been established yet.

In this research the methods used allowed an accurate and rapid measurement of total anthocyanin content (TAC) and total polyphenolic content (TPC). The TPC, 194.22 ± 32.83 mg of gallic acid equivalents (GAE) on 100 g of fresh weight (FW), and TAC, 147.42 ± 0.58 mg of cyanidin 3-*O*-glucoside (C3G) on 100 g_FW_, values obtained from the analyzed extracts (Table 1) were similar to values reported by Kucharska and Oszmianski [5] and Pasko et al. [1]: in these and other similar studies, TAC in *P. padus* ranged from 190 to 281 mg_C3G_/100 g_FW_, while TPC ranged from 60 to 370 mg_GAE_/100 g_FW_. Several internal and external factors such as genetic variability as well as many climatic and environmental conditions (light intensity, humidity, temperature, the use of specific agrotechniques, infections or other stress factors) could determine the differences in phenolic and anthocyanin [11].

Antioxidant bioactive compounds may act as reducing agents, hydrogen donators, and singlet oxygen quenchers thanks to their redox properties. In this research, the ferric reducing antioxidant power (FRAP) assay, based on the ability of antioxidants to reduce ferric(III) ions to ferrous(II) ions, was used to evaluate antioxidant capacity of bird cherry fruits. Although the FRAP protocol is an in vitro chemical-based assay without large application in biological systems (antioxidant action is not limited to scavenging free radicals but includes up-regulation of antioxidant and detoxifying enzymes, modulation of redox cell signaling and gene expression), many studies showed that it may highlight the potential of a sample as inhibitor of a target molecule oxidation: this chemical-based method is useful for screening, it is low cost, high throughput and yields an index value (expressed as ferrous iron equivalents) that allows comparing and ordering different samples in order to sense the antioxidant power of natural products [12,13]. In any case, there are many oxidative stress biomarkers to investigate, and therefore, to define the antioxidant and nutraceutical properties of a food as shown by Frijhoff et al. [14]. Fresh fruits showed a mean FRAP value of 17.78 ± 0.84 mmol Fe^2+^·kg^−1^ (Table 1), according to other studies [1]. Moreover, FRAP assay showed a FRAP value similar to raspberry, orange, and black mulberry [6,7], but lower than other common berry fruits as blueberry, blackcurrant, and blackberry [15]. Different bioactive compounds contribute to the antioxidant activity of fruit extracts. For example, the antioxidant capacity of some polyphenols, as anthocyanins, is higher than vitamin C activity: antioxidant activity of cyanidin is 4–4.5 times higher than ascorbic acid [10]. In this study, the correlation between TPC/TAC and antioxidant activity was strongly positive, with significant Pearson correlation coefficients (R = 0.938 for TPC and R = 0.998 for TAC).

### 2.3. Phytochemical Composition

Antioxidant compounds (polyphenols and vitamin C), terpenes, and organic acids may be nutritionally important and play an health-promoting critical role in humans for disease prevention due to their synergistic or additive biological effects (phytocomplex) [15]. The current practice for chemical characterization is carried out measuring the quantity of the main bioactive compounds, called ‘‘markers’’: in this study 28 biologically active compounds were selected as markers for fingerprint analysis because they have been described as important health-effective substances in humans [15].

This study contributed to the knowledge of the phytochemical composition and antioxidant properties of the bird cherry fruits, supporting a larger exploitation of this fruit tree-species. In particular, these fruits could be useful for the food industry, as a potential natural source of bioactive compounds with high antioxidant properties, due to their contents of organic and phenolic acids, catechins, flavonoids, and vitamin C.

The chemical fingerprint of *P. padus* fruits is reported in Table 2 and Table 3 (polyphenolic compounds and other health-promoting agents, respectively). The total bioactive compound content (TBCC) was calculated as the sum of the main molecules selected for their healthy properties and detected in the extracts: results showed a mean TBCC value of 1139.23 ± 19.15 mg/100 g_FW_.

Synergistic and additive antioxidant effects of bioactive compounds were evaluated via FRAP assay as preliminary test: Pearson’s correlation coefficient (R) between antioxidant activity and the other phytochemical parameters was used to assess the correlation between each bioactive class and total antioxidant activity as reported in Table 4.

A preliminary phytochemical fingerprint of bird cherry fruits was described. 23 bioactive compounds were separated and identified via HPLC-DAD: adding other markers with demonstrated biological activity would be a necessary step for a better identification of the chromatographic pattern in further fingerprint studies together with a mass spectrometry detection of unknown peaks. In particular, polyphenolic compounds detected in these fruits have not yet been fully identified in literature: liquid chromatography coupled to mass/mass spectrometry (LC–MS/MS) is a very effective technique for complex plant extract analysis providing a rapid and accurate identification of phytochemicals, as phenolics [16,17]. Mass/mass spectrometry data of selected and identified polyphenols in *P. padus* fruits were reported in supplementary materials (Table A1). *P. padus* fruits showed the following bioactive compound composition: four cinnamic acids (caffeic, chlorogenic, coumaric, and ferulic acids), four flavonols (hyperoside, quercetin, quercitrin, rutin), two benzoic acids (ellagic and gallic acids), two catechins ((+)catechin, (−)epicatechin), two tannins (castalagin, vescalagin), four monoterpenes (limonene, phellandrene, sabinene, γ-terpinene), three organic acids (citric, oxalic, and quinic acids), and vitamin C (expressed as the sum of ascorbic and dehydroascorbic acids). Isoquercitrin, terpinolene, malic acid, succinic acid, and tartaric acid were not detected, in agreement with *P. padus* fruit phytochemical fingerprints reported in similar studies [1,5].

The single contribution of each class to total fruit phytocomplex was evaluated grouping the identified health-promoting molecules into bioactive classes. The most important class was organic acids (48.62 ± 2.31%), followed by polyphenols (35.34 ± 1.80%) as the sum of anthocyanins, phenolic acids, flavonols, catechins and tannins, monoterpenes (9.36 ± 0.64 5%), and vitamin C (6. 68 ± 0.22%) (mean values were considered).

Therefore, organic acids represented the first component of the *P. padus* fruit fingerprint (553.88 ± 51.27 mg/100 g_FW_) as already reported by Pasko et al. [1]. These compounds, in particular citric acid (217.24 ± 14.95 mg/100 g_FW_) and quinic acid (324.48 ± 57.21 mg/100 g_FW_), are mainly used to determine fruit maturity stage because they affect some fruit quality properties (e.g., stability, color, and flavor) indicating the spoilage of fruit products [8], but they are also antioxidants with multi-purpose uses in pharmacology as reported by Eyduran et al. [18]. These molecules are also used as chemical markers to identify adulteration in fruit juices and considered as food acidifiers by food industry.

**Figure 1:**
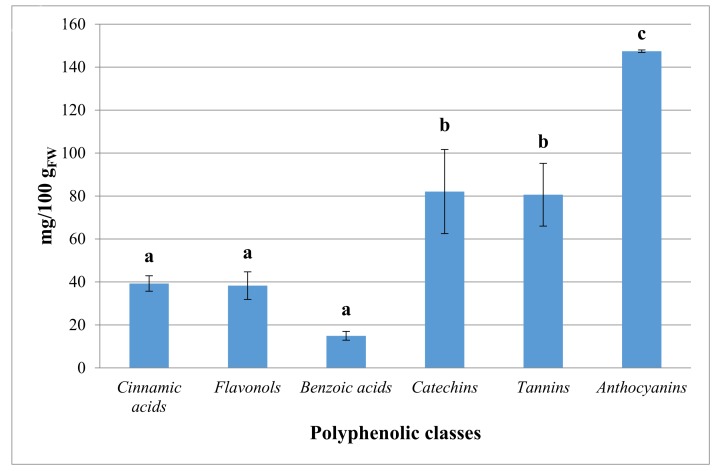
Polyphenolic composition in bird cherry fruits. Mean value and standard deviation (SD) of each polyphenolic class is given (N = 3). Different letters for each class indicate the significant differences at *p* < 0.05. Results are expressed as mg/100 g_FW_ (FW = fresh weight).

**Figure 2:**
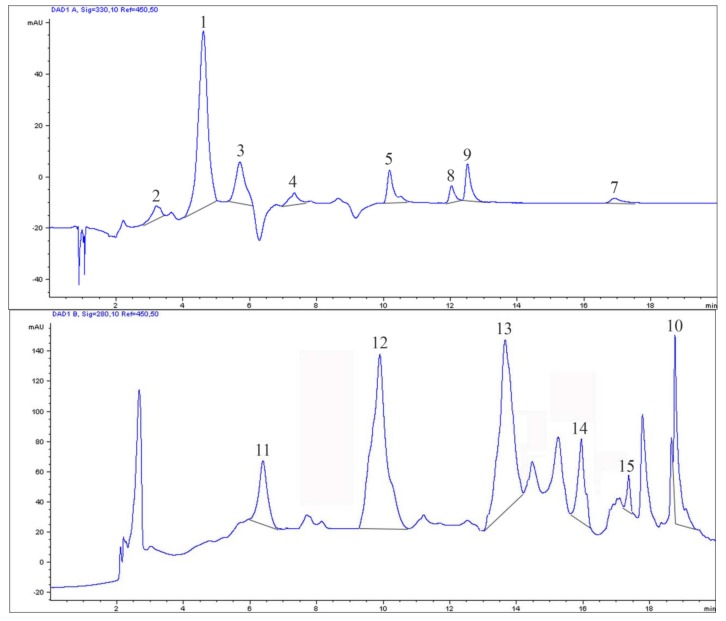
HPLC fingerprint of selected polyphenolic compounds with high health-promoting properties. For peak identification: caffeic acid—1, chlorogenic acid—2, coumaric acid—3, ferulic acid—4, hyperoside—5, isoquercitrin—6, quercetin—7, quercitrin—8, rutin—9, ellagic acid—10, gallic acid—11, (+)catechin—12, (−)epicatechin—13, castalagin—14, and vescalagin—15. Isoquercitrin (6) was searched but not detected in the extracts.

**Figure A1:**
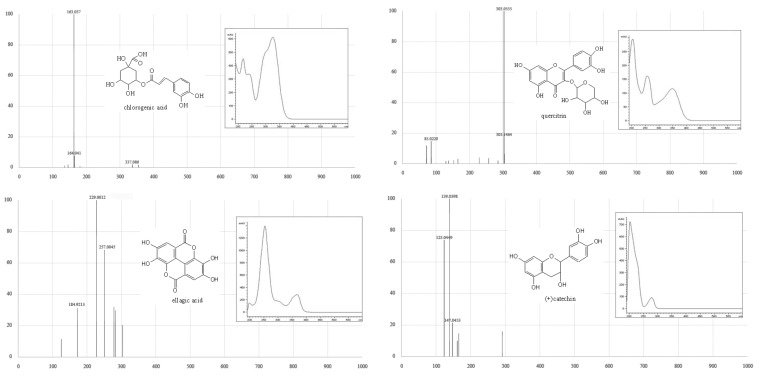
Chemical structure and spectroscopic information of 4 polyphenolic compounds selected for their high health-promoting properties.

Moreover, results showed that bird cherry fruits could be a good source of phenolic constituents and deserved special attention focused on studying their phytochemical profile as reported in other studies on different genotypes [1]: the most important polyphenolic classes (Figure 1) were anthocyanins (147.42 ± 0.58 mg/100 g_FW_) > catechins (82.09 ± 19.58 mg/100 g_FW_) > tannins (80.61 ± 14.61 mg/100 g_FW_) > cinnamic acids (39.29 ± 3.59 mg/100 g_FW_) > flavonols (38.29 ± 6.43 mg/100 g_FW_) > benzoic acids (14.94 ± 2.05 mg/100 g_FW_). In this study, the correlation between polyphenolic class and antioxidant activity was positive, with a significant Pearson correlation coefficient (R = 0.682). HPLC fingerprint of identified polyphenols in *P. padus* fruits was reported in Figure 2. For many years biological protective effects of polyphenols have been often ascribed mainly to their antioxidant activity [19]: however, more complex actions are now being considered [20]. Plants containing flavonoids are often used to treat diabetes in several natural medicines; these compounds show glucose-lowering effects repressing hepatic glucose production in animals [21]. Flavonoids are polyphenolic compounds comprising 15 carbons with two aromatic rings connected by a three-carbon bridge and they occur widely throughout the plant kingdom with the exception of fungi and algae. In particular, flavonols show the potential to quench active oxygen species and inhibit in vitro oxidation of low-density lipoproteins reducing thrombotic tendency. Flavonols may inhibit cyclooxygenase modulating arachidonic acid metabolism and attenuating inflammation. Many natural flavonoids have also shown anti-carcinogenic activity in several animal models. In addition, they may interact with carcinogens in the gastrointestinal tract reducing their adsorption [22]. In the flavonoid group, flavonols may exert critical beneficial features related to human health. In this research the most important flavonols selected as marker were quercitrin (16.37 ± 3.51 mg/100 g_FW_) and quercetin (11.86 ± 2.36 mg/100 g_FW_), followed by hyperoside (7.38 ± 0.41 mg/100 g_FW_) and rutin (2.67 ± 1.02 mg/100 g_FW_), according to previous studies [1]. Moreover, the presence of cinnamic acids, in particular chlorogenic acid (10.48 ± 0.28 mg/100 g_FW_), may be important when fruits are processed, as these compounds are considered a preferential substrate for the catecholase activity of polyphenol oxidase [23]. Cinnamic acids, as caffeic, ferulic and coumaric acids, could also influence the oxidation and color development processes occurring during technological processing [24]. As previously discussed, anthocyanins (147.42 ± 0.58 mg/100 g_FW_) are of prominent importance in *P. padus* fruits for two reasons: they constitute an important part of the quality traits, because their levels directly pertain to the fruit coloration, and, on the other hand, they have been claimed to possess several biological properties and an high potential nutraceutical value [25]. Ellagic (11.41 ± 1.25 mg/100 g_FW_) and gallic acids (3.54 ± 0.81 mg/100 g_FW_) were also detected in *P. padus* extracts: these molecules are very important in human nutrition and are related to many biological properties, including, anticancer, anti-atherosclerotic, anti-inflammatory, antihepatotoxic, and anti-HIV replication activities [26]. The presence of tannins in adequate amounts (80.61 ± 14.61 mg/100 g_FW_) in bird cherry extracts are advantageous as they are able to very effectively quench free radicals [27]. The identification of (+)catechin (56.66 ± 16.88 mg/100 g_FW_) and (−)epicatechin (25.43 ± 3.16 mg/100 g_FW_) is an important result as they are involved in the inhibition of lipid peroxidation, and inhibition of human cancer cell line proliferation and cyclooxygenase enzymes as similar compounds belonging to the bioactive class of catechins [28]. In Figure A1 spectroscopic information of 4 polyphenolic compounds (chlorogenic acid, quercitrin, ellagic acid, and (+)catechin) with high demonstrated health-promoting properties were reported.

Monoterpenes represent an important fraction of the TBCC of bird cherry fruits (106.65 ± 7.51 mg/100 g_FW_): the plant terpenoids are potential health-promoting groups because they are a large class of naturally bioactive molecules used extensively for their aromatic qualities together with their antioxidant capacity and anti-inflammatory properties [29]. Monoterpenes are non-nutritive dietary components found in the essential oils of several plants. An high number of these molecules have antibacterial and antitumor activity [30]. In this study, γ-terpinene (65.52 ± 6.25 mg/100 g_FW_) and limonene (31.40 ± 5.65 mg/100 g_FW_) were the most abundant monoterpenes: several studies reported their chemopreventive activity against rodent mammary, skin, liver, lung and forestomach cancers [31].

Vitamin C was evaluated as the sum of ascorbic and dehydroascorbic acids (25.20 ± 3.48 mg/100 g_FW_ and 50.87 ± 16.23 mg/100 g_FW_, respectively) due to their biological activity in human organisms [32]. Bird cherry fruits showed a total vitamin C content (76.07 ± 13.34 mg/100 g_FW_) similar to the most common fruits, as kiwifruit (74.56 ± 9.84 mg/100 g_FW_) and orange (71.12 ± 1.96 mg/100 g_FW_), as previously reported by several pieces of research [15]. For this reason, a portion of 100 g of bird cherry fruits may contribute to the recommended daily intake of vitamin C (60–90 mg) [33]. However, the use of bird cherry fresh fruits as functional food is limited by their slightly more bitter taste if compared to other common fruit species [2].

## 3. Materials and Methods

### 3.1. Plant Material, Harvesting Site and Pedoclimatic Information

Fully ripened fruits (0.5 kg for each biological replication) of a cultivated genotype of *Prunus padus* L. (Colorata cv) were collected in the in mid-July 2017, in Chieri (45°1′0′′ N, 7°49′0′′ E, at 305 m A.S.L.), Piedmont (north-western Italy), in a germplasm repository of the Department of Agricultural, Forest and Food Sciences, University of Turin (voucher specimens were deposited). Fruits were manually harvested from the plants based on selected qualitative parameters (firmness and total soluble solids), considering also literature and experience of the University researchers. The climate of the area is temperate, with rains in spring and autumn, and average precipitation of approximately 810 mm/year; the soil is loam–clay.

The fruits were selected, having removed those with cuts or those damaged by insects, and then divided into two equal portions. One portion was used to determine their morphological and physico-chemical parameters, on the same day of harvest. The second portion was stored at 4 °C and 95% relative humidity (RH) for one day and then used for phytochemical extraction.

### 3.2. Solvents and Chemicals

Sodium carbonate, Folin-Ciocalteu phenol reagent, sodium acetate, citric acid, potassium chloride, hydrochloric acid, iron(III) chloride hexahydrate, 2,4,6-tripyridyl-*S*-triazine (TPTZ), 1,2-phenylenediamine dihydrochloride (OPDA), all polyphenolic (caffeic acid, chlorogenic acid, coumaric acid, ferulic acid, hyperoside, isoquercitrin, quercetin, quercitrin, rutin, ellagic acid, gallic acid, (+)catechin, (−)epicatechin, castalagin, and vescalagin) and terpenic standards (limonene, phellandrene, sabinene, γ-terpinene, and terpinolene), potassium dihydrogen phosphate, phosphoric acid and HPLC-grade methanol and acetonitrile were purchased from Sigma-Aldrich (St. Louis, MO, USA). Ethylenediaminetetraacetic acid disodium salt was purchased from AMRESCO (Solon, OH, USA). Sodium fluoride was purchased from Riedel-de Haen (Seelze, Germany). Milli-Q ultrapure water was produced by Sartorius Stedim Biotech mod. Arium (Sartorius, Göttingen, Germany). Cetyltrimethylammonium bromide (cetrimide), ascorbic acid (AA) and dehydroascorbic acid (DHAA) were purchased from Extrasynthése (Genay, France). Acetic acid, ethanol, organic acids (citric acid, malic acid, oxalic acid, quinic acid, succinic acid, and tartaric acid) and HPLC-grade formic acid were purchased from Fluka BioChemika, Buchs, Switzerland.

### 3.3. Determination of Quality Properties

The fruit width and length were measured using a 0.01 mm sensitive digital caliper (Traceable Digital Caliper-6′′, VWR International, Milano, Italy) and their weight determined to the nearest 0.01 g (Mettler, Greifensee, Switzerland). The fruit without kernel was then homogenized in a blender and centrifuged (4000 rpm, 10 min) to separate solid parts from juice, and pH, total soluble solids (TSS), and titratable acidity (TA) were evaluated.

The TSS was measured with a digital refractometer (Tsingtao Unicom-Optics Instruments, Laixi, China) and results were expressed as °Brix. The TA (meq·L^−1^) was determined by titration of a mixture of bird cherry juice (10 mL) diluted in Milli-Q water (90 mL) with a 0.2 M NaOH solution using an automatic titrator (Crison, Alella, Spain) to an end-point of pH 8.2. The pH of the fruit juice was directly measured with the aid of a potentiometric pH-meter (Crison, Alella, Spain).

### 3.4. Bioactive Compound Extraction

#### 3.4.1. Polyphenolic Compounds

For the extraction of bioactive compounds, 10 g of fruit without kernel (three replications) were put into a 50-mL test tube and 25 mL of extraction solution (methanol:bi-distilled water, 95:5 *v*/*v*, pH adjusted with 1.5 mL of 37% HCl) and were then added to the weighed samples. After 60 min in the dark, the extracts were homogenized with an Ultra-Turrax (IKA-Werkemodel T25, Staufen, Germany) for about 1 min and then centrifuged for 15 min at 3000 rpm in a Centrifuge (ALC Centrifuge model PK 120, Cologno Monzese, Italy). This operation was carried out 3 times. All the supernatants were recovered and transferred to small glass tubes and kept frozen at −20 °C for further analysis.

#### 3.4.2. Organic Acids and Monoterpenes

For the extraction of organic acids and monoterpenes, three replications were considered. Five grams of fruit without kernel were put into a test tube and 25 mL of 95% ethanol solution were then added. After 30 min in the dark, the extracts were homogenized with an Ultra-Turrax (T25, IKA WERKE) for about 1 min and then centrifuged for 10 min at 4000 rpm in an ALC Centrifuge PK 120 (ALC International, Cologno Monzese, Italy). This operation was carried out 2 times. All the supernatants were recovered and transferred to small glass tubes and kept frozen at −20 °C for further analysis.

#### 3.4.3. Vitamin C

A total of 10 g of fruit without kernel (three replications) was put into a 50-mL test tube and 10 mL of extraction solution (0.1 M citric acid, 2 mM EDTA disodium salt and 4 mM sodium fluoride in methanol–water, 5:95 *v*/*v*) were then added. The extracts were homogenized with an Ultra-Turrax (IKA-Werke T25) for about 1 min and then centrifuged for 10 min at 4000 rpm at room temperature in an ALC Centrifuge PK 120. The supernatants were recovered and transferred to a 15-mL test tube through filter cloth and then acidified with 4 N HCl to decrease pH solution to a value of 2.2–2.4 pH units. Acidified samples were centrifuged for 5 min at 12,000 rpm at 4 °C with an ALC Multispeed refrigerated centrifuge PK 121R (ALC International, Cologno Monzese, Italy) [34].

### 3.5. Spectrophotometric Analysis

The total polyphenol content (TPC) was determined using the Folin-Ciocalteu assay [35], reporting results as mg of gallic acid equivalents (GAE) per 100 g of fresh weight (FW). A total of 0.5 g of fruit extract and 30 mL of bi-distilled water was added to 2.5 mL of Folin–Ciocalteu reagent and 10 mL of Na_2_CO_3_ 15%; after 2 h in the dark, absorbance was 765 nm. The standard calibration curve was plotted using gallic acid at concentrations of 0.02–0.1 mg/mL.

The total anthocyanin content (TAC) was obtained using the pH-differential method [36], and expressed as milligrams of cyanidin 3-*O*-glucoside (C3G) per 100 gm of fresh weight (mg_C3G_/100 g_FW_). A molar absorptivity of 26,900 was used for cyanidin 3-*O*-glucoside (molecular weight 449.2). Extracts in 0.025 M potassium chloride buffer (pH 1.0) and 0.4 M sodium acetate buffer (pH 4.5) were measured at 510 and 700 nm after 20 min of incubation at 23 °C. Anthocyanins demonstrate maximum absorbance at 515 nm at pH 1.0 and at 700 nm at pH 4.5. The colored oxonium form of anthocyanin predominates at pH 1.0, and the colorless hemiketal form at pH4.5. The pH-differential method is based on the reaction producing oxonium forms.

For the evaluation of antioxidant activity the ferric reducing antioxidant power (FRAP) assay [37] was used and results were expressed as millimoles of ferrous iron (Fe^2+^) equivalents per kilogram (solid food) of FW. The method is based on the reduction of the ferric (Fe^3+^) 2,4,6-tris(2-pyridyl)-s-triazine (TPTZ) complex to its ferrous form (Fe^2+^). The FRAP reagent was prepared daily by mixing a TPTZ solution and a FeCl_3_·6H_2_O solution with acetate buffer (0.3 M), and then warmed at 37 °C before using; 30 µL of sample (15 µL of extracted sample and 15 µL of extraction buffer, dilution 1:2) was added to 90 µL of bi-distilled water and 900 µL of FRAP reagent in a 2-mL microtube and then incubated at 37 °C for 30 min in a Shaking Water Bath (G.F.L. Shaking Water Bath mod.1083, Burgwedel, Germany). Absorbance was read at 595 nm. Standard curve was obtained using FeSO_4_·7H_2_O (concentration range: 100–1000 mmol/L).

### 3.6. Chromatographic Analysis

#### 3.6.1. Sample Preparation Protocols for HPLC Analysis

Samples were filtered with circular pre-injection filters (0.45 µm, polytetrafluoroethylene membrane) prior to HPLC-DAD analysis. In the case of vitamin C analysis, a C_18_ cartridge for solid phase extraction (Sep-Pak^®^ C-18, Waters, Milford, MA, USA) was used to separate the polyphenolic fraction from vitamin C (ascorbic acid–AA plus dehydroascorbic acid–DHAA). Then, *o*-phenylenediamine (OPDA) solution (18.8 mmol·L^−1^) was added to each sample for DHAA derivatization into the fluorophore 3-(1,2-dihydroxyethyl)furo(3,4-*b*)quinoxaline-1-one. After 37 min in the dark, these samples were analyzed using HPLC-DAD [34].

#### 3.6.2. Apparatus and Chromatographic Conditions

An Agilent 1200 High-Performance Liquid Chromatograph coupled to an Agilent UV-Vis diode array detector (Agilent Technologies, Santa Clara, CA, USA) was used for the chromatographic analysis.

Bioactive substances were separated on a Kinetex C18 column (4.6 × 150 mm, 5 μm, Phenomenex, Torrance, CA, USA). Five different chromatographic methods were used to analyze the samples, two for polyphenols and one for terpenic compounds, organic acids, and vitamins, respectively [9]. Several mobile phases were used for compound separation and identification and UV spectra were recorded at different wavelengths, as listed in Table 5.

The chromatographic conditions were set to obtain a phytochemical fingerprint containing compositional information with a good resolution and a reasonable analysis time. Different linear gradients in different slopes were used for optimizing the molecule separation because some compounds were similar in structure with each other in the same chemical class. Formic and phosphoric acid was added for enhancing the resolution and eliminating peak tailing because most of the compounds were also weakly acidic. Selected wavelengths were suitable to achieve more specific peaks as well as a smooth baseline after a full-scan on the chromatogram from 190 to 400 nm.

#### 3.6.3. Identification and Quantification of Bioactive Compounds in the Extracts

External standard calibration method was used for quantitative determinations by plotting the peak area (*y*) of the compound versus the sample concentration (*x*): for each analytical standard: three manual injections (20 µL) at each concentration were performed. The approach for accounting for matrix effects was to build a calibration curve using standard samples with known analyte concentration approximating the matrix of the sample as much as possible. The limit of detection (LOD) and the limit of quantification (LOQ) of the used chromatographic methods, defined as the lowest amount of analyte that gives a reproducible peak with a signal to-noise ratio (S/N) of 3 and 10 respectively, were reported in Table 6. All the samples were analyzed in triplicate, and the results were reported as means ± standard deviation (SD) to assess the repeatability of the used methods. Accuracy was checked by spiking samples with a solution containing each bioactive compound in a concentration of 10 mg·mL^−1^.

In this preliminary study, compounds were identified by the comparison of the retention times (t_R_) and maximum absorbance values of detected peaks in samples with those obtained by injection of pure standards in the same matrix conditions. Total bioactive compound content (TBCC) was determined as the sum of selected markers having a positive role in human health as reported in the “multi-marker approach” by Mok and Chau [38]. Five polyphenolic classes were considered: benzoic acids, catechins, cinnamic acids, flavonols, and tannins. Monoterpenes, organic acids, and vitamin C (as the sum of ascorbic and dehydroascorbic acids) were also considered to obtain an analytical fingerprint. All the results were expressed as mg/100 g of FW (fresh weight).

### 3.7. Statistical Analysis

Analysis of variance was performed by one-way ANOVA analysis (SPSS 22.0) followed by Tukey’s HSD post hoc comparison test at *p* < 0.05 (N = 3). Correlation between antioxidant activity and the other phytochemical parameters was evaluated with Pearson’s coefficient (R) at *p* < 0.05 (N = 3).

## 4. Conclusions

The results revealed that European bird cherry fruit is rich in anthocyanins and other health-promoting agents, similarly to berry and other common fruits reported as antioxidant rich sources, and particularly useful as fresh functional foods or fruit-derived products (e.g., juices, fruit drinks, and wines). It is worth emphasizing that bioactive compounds from bird cherry are not only important components for cheap natural food suitable for consumption, but they could also be valuable substances for pharmaceutical products due to their antioxidative potential. For these reasons, the determination of the natural antioxidant compounds from *P. padus* fruits may help develop at least a potential template for functional food candidates for antioxidant therapy.

Additional research should be carried out to improve knowledge regarding the health benefits, antioxidant activity, and phytochemical profile of *P. padus*. Specific cultivars should be harvested and included in food products and daily nutrition for their high healthy properties as potential functional foods.

## Figures and Tables

**Table 1:** Quality and nutraceutical traits of bird cherry fruits.

Traits	Parameters	Mean Value ± SD
Quality	Weight ^a^	2.08 ± 0.02
	Width ^b^	11.94 ± 0.09
	Length ^c^	11.11 ± 0.08
	Total soluble solids ^d^	16.17 ± 0.45
	Titratable acidity ^e^	245.33 ± 13.32
	pH ^f^	2.93 ± 0.03
Nutraceutical	Total polyphenolic content ^g^	194.22 ± 32.83
	Antioxidant activity ^h^	17.78 ± 0.84
	Total anthocyanin content ^i^	147.42 ± 0.58

Mean value and standard deviation (SD) of each sample is given (N = 3). Results were expressed as: ^a^ g; ^b^ mm; ^c^ mm; ^d^ Brix; ^e^ meq·L^−1^; ^f^ pH-units; ^g^ mg_GAE_/100 g_FW_; ^h^ mmol Fe^2+^/kg; ^i^ mg_C3G_/100 g_FW_.

**Table 2:** Phytochemical fingerprint of the polyphenolic compounds found in bird cherry fruits.

Polyphenolic Class	Compound	Mean Value	SD
Cinnamic acids	caffeic acid	6.16	1.35
	chlorogenic acid	10.48	0.28
	coumaric acid	12.20	3.07
	ferulic acid	10.45	3.65
Flavonols	hyperoside	7.38	0.41
	isoquercitrin	n.d.	/
	quercetin	11.86	2.36
	quercitrin	16.37	3.51
	rutin	2.67	1.02
Benzoic acids	ellagic acid	11.41	1.25
	gallic acid	3.54	0.81
Catechins	(+)catechin	56.66	16.88
	(−)epicatechin	25.43	3.16
Tannins	castalagin	53.95	8.90
	vescalagin	26.66	5.97

Mean value and standard deviation (SD) of each sample is given (N = 3). Results are expressed as mg/100 g_FW_ (FW = fresh weight).

**Table 3:** Phytochemical fingerprint (vitamin C and other health-promoting molecules) of bird cherry fruits.

Bioactive Class	Compound	Mean Value	SD
Monoterpenes	limonene	31.40	5.65
	phellandrene	8.51	2.69
	sabinene	1.21	0.18
	γ-terpinene	65.52	6.25
	terpinolene	n.d.	/
Organic acids	citric acid	217.24	14.95
	malic acid	n.d.	/
	oxalic acid	12.16	2.19
	quinic acid	324.48	57.21
	succinic acid	n.d.	/
	tartaric acid	n.d.	/
Vitamin C	ascorbic acid	25.20	3.48
	dehydroascorbic acid	50.87	16.23

Mean value and standard deviation (SD) of each sample is given (N = 3). Results are expressed as mg/100 g_FW_ (FW = fresh weight).

**Table 4:** Correlation between antioxidant activity and the other phytochemical parameters in analyzed fruits by Pearson’s correlation coefficient (R).

R	Pearson’s Correlation Coefficient					
	TPC	TAC	Polyphenols	Monoterpenes	Organic Acids	Vitamin C
**Antioxidant activity**	0.937	0.998	0.682	0.808	0.437	0.361

Correlation was evaluated at *p* < 0.05 (N = 3).

**Table 5:** Chromatographic conditions of the used methods.

Method	Compounds of Interest	Mobile Phase	Flow (mL min^−1^)	Wavelength (nm)
A	cinnamic acids, flavonols	A: 10 mM KH_2_PO_4_/H_3_PO_4_	1.5	330
		B: CH_3_CN		
B	benzoic acids, catechins, tannins	A: H_2_O/CH_3_OH/HCOOH (5:95:0.1 *v*/*v*/*v*)	0.6	280
		B: CH_3_OH/HCOOH (100:0.1 *v*/*v*)		
C	monoterpenes	A: H_2_O	1.0	210, 220,
		B: CH_3_CN		235, 250
D	organic acids	A: 10 mM KH_2_PO_4_/H_3_PO_4_	0.6	210, 214
		B: CH_3_CN		
E	vitamin C	A: 5 mM C_16_H_33_N(CH_3_)_3_Br/50 mM KH_2_PO_4_	0.9	261, 348
		B: CH_3_OH		

*Elution conditions:* Method A: gradient analysis: 5%B to 21%B in 17 min + 21%B in 3 min (2 min conditioning time); Method B: gradient analysis: 3%B to 85%B in 22 min + 85%B in 1 min (2 min conditioning time); Method C: gradient analysis: 30%B to 56%B in 15 min + 56%B in 2 min (3 min conditioning); Method D: gradient analysis: 5%B to 14%B in 10 min + 14%B in 3 min (2 min conditioning time); Method E: isocratic analysis: ratio of phase A and B: 95:5 in 10 min (5 min conditioning time).

**Table 6:** Calibration parameters for all the used analytical standards.

Class	Standard	t_R_^a^ (min)	Calibration Curve Equation	R^2^	Calibration Curve Range (mg L^−1^)	Wavelength (nm)	LOD ^b^ (mg L^−1^)	LOQ ^c^ (mg L^−1^)
Cinnamic acids	caffeic acid	4.54	y = 59.046x + 200.6	0.996	111–500	330	0.305	1.016
	chlorogenic acid	3.89	y = 13.583x + 760.05	0.984			0.940	3.134
	coumaric acid	5.74	y = 8.9342x + 217.4	0.997			2.907	9.690
	ferulic acid	7.99	y = 3.3963x − 4.9524	1.000			1.245	4.150
Flavonols	hyperoside	10.89	y = 7.1322x − 4.583	0.999	111–500	330	3.372	11.241
	isoquercitrin	11.24	y = 8.3078x + 26.621	0.999			0.252	0.840
	quercetin	17.67	y = 3.4095x − 98.307	0.998			4.055	13.518
	quercitrin	13.28	y = 2.7413x + 5.6367	0.998			5.456	18.187
	rutin	12.95	y = 6.5808x + 30.831	0.999			2.937	9.790
Benzoic acids	ellagic acid	18.65	y = 29.954x + 184.52	0.998	62.5–250	280	0.611	2.035
	gallic acid	6.26	y = 44.996x + 261.86	0.999			0.435	1.451
Catechins	(+)catechin	10.31	y = 8.9197x + 66.952	1.000	62.5–250	280	2.343	7.809
	(−)epicatechin	14.30	y = 12.88x − 43.816	0.999			0.763	2.543
Tannins	castalagin	16.35	y = 4.236x − 8.535	1.000	62.5–250	280	1.009	3.363
	vescalagin	17.25	y = 4.939x − 1.232	1.000			0.603	2.010
Monoterpenes	limonene	3.35	y = 0.1894x − 5.420	0.999	125–1000	250	8.654	28.847
	phellandrene	4.85	y = 8.783x − 145.3	0.998		210	0.562	1.874
	sabinene	3.65	y = 18.14x − 1004	0.998		220	0.094	0.314
	γ-terpinene	5.28	y = 0.4886x − 23.02	0.999		235	17.577	58.590
	terpinolene	4.83	y = 26.52x + 876.8	0.999		220	0.241	0.804
Organic acids	citric acid	5.30	y = 1.0603x − 22.092	1.000	167–1000	210, 214	18.805	62.682
	malic acid	4.03	y = 1.415x − 80.254	0.996			15.721	52.404
	oxalic acid	7.85	y = 6.4502x + 6.1503	0.998			0.550	1.835
	quinic acid	3.21	y = 0.8087x − 38.021	0.998			26.106	87.021
	succinic acid	3.46	y = 0.9236x − 8.0823	0.995			7.135	23.783
	tartaric acid	5.69	y = 1.8427x + 15.796	1.000			8.520	28.401
Vitamin C	ascorbic acid	5.03	y = 42.71x + 27.969	0.999	100–1000	261	0.836	2.786
	dehydroascorbic acid	3.30	y = 4.1628x + 140.01	0.999	30–300	348	1.095	3.649

^a^ t_R_ = retention time; ^b^ LOD = limit of detection; ^c^ LOQ = limit of quantification.

## Appendix A

**Table A1:** Mass/mass spectrometry data of the polyphenolic compounds detected in bird cherry fruits.

Class	Compound	Ionization Mode ^a^	Ionization Type ^b^	Parent Ion ^c^	MS^2^ Ions ^d,e^	Collision Energy ^f^ (eV)
				(*m*/*z*)		
Cinnamic acids	caffeic acid	positive	ESI	181.16	**89**, 117	30
	chlorogenic acid	positive	ESI	355.31	**163**, 164, 337	10
	coumaric acid	positive	ESI	165.16	**147**, 119, 91	5–60 (ramp)
	ferulic acid	positive	ESI	195.18	**195**, 177, 145	30
Flavonols	hyperoside	positive	ESI	465.38	**303**, 304	30
	isoquercitrin	positive	ESI	465.38	**303**, 304, 127	30
	quercetin	positive	ESI	303.26	**303**, 304, 257	35
	quercitrin	positive	ESI	435.35	**303**, 85	30
	rutin	positive	ESI	611.52	**303**, 304, 465	30
Benzoic acids	ellagic acid	negative	ESI	301.19	**229**, 257, 184	30
	gallic acid	negative	ESI	169.12	**79**, 51, 67	50
Catechins	(+)catechin	positive	ESI	291.27	**139**, 123, 147	5–60 (ramp)
	(−)epicatechin	positive	ESI	291.27	**139**, 123, 165	35
Tannins	castalagin	negative	ESI	933.63	**301**, 631, 915	50
	vescalagin	negative	ESI	933.63	**301**, 613, 915	50

^a^ Mass spectra were acquired operating in both positive [M − H]^+^ and negative [M − H]^−^ ionization modes (Quadrupole time-of-flight mass spectrometer—QTOF); ^b^ ESI source was chosen instead of APCI (Atmospheric Pressure Chemical Ionization) and APPI (Atmospheric Pressure Photoionization) sources as the phenolic compounds were small and relatively polar molecules; ^c^ Parent ion (*m*/*z*): molecular ions of the standard compounds (mass to charge ratio); ^d^ MS^2^: fragments for the related molecular ions. Tandem mass spectrometry was used due to its fragmented ion stability; ^e^ Most intense signal/s in MS/MS spectra is/are highlighted in bold; ^f^ Collision energy (CE) refers to related collision energies of the fragment ions.

## References

[1] Pasko P., Makowska-Was J., Chlopicka J., Szlosarczyk M., Tyszka-Czochara M., Dobrowolska-Iwanek J., Galanty A. (2012). South siberian fruits: Their selected chemical constituents, biological activity, and traditional use in folk medicine and daily nutrition. J. Med. Plants Res..

[2] Leather S.R. (1996). *Prunus padus* L. J. Ecol..

[3] Lenchyk L.V., Upyr D.V., Ovezgeldiyev D. (2016). Phytochemical investigation of bird cherry fruits. Der Pharmacia Lettre.

[4] Uusitalo M. (2004). European Bird Cherry (Prunus padus *L*.)—A Biodiverse Wild Plant for Horticulture.

[5] Kucharska A.Z., Oszmianski J. (2002). Anthocyanins in fruits of *Prunus padus* (bird cherry). J. Sci. Food Agric..

[6] Donno D., Cavanna M., Beccaro G.L., Mellano M.G., Torello-Marinoni D., Cerutti A.K., Bounous G. (2013). Currants and strawberries as bioactive compound sources: Determination of antioxidant profiles with HPLC-DAD/MS. J. Appl. Bot. Food Qual..

[7] Canterino S., Donno D., Mellano M.G., Beccaro G.L., Bounous G. (2012). Nutritional and sensory survey of *Citrus sinensis* (L.) cultivars grown at the most northern limit of the mediterranean latitude. J. Food Qual..

[8] Mikulic-Petkovsek M., Rescic J., Schmitzer V., Stampar F., Slatnar A., Koron D., Veberic R. (2015). Changes in fruit quality parameters of four ribes species during ripening. Food Chem..

[9] Donno D., Cerutti A.K., Mellano M.G., Prgomet Z., Beccaro G.L. (2016). Serviceberry, a berry fruit with growing interest of industry: Physicochemical and quali-quantitative health-related compound characterisation. J. Funct. Foods.

[10] Veberic R., Slatnar A., Bizjak J., Stampar F., Mikulic-Petkovsek M. (2015). Anthocyanin composition of different wild and cultivated berry species. LWT Food Sci. Technol..

[11] Heimler D., Romani A., Ieri F. (2017). Plant polyphenol content, soil fertilization and agricultural management: A review. Eur. Food Res. Technol..

[12] López-Alarcón C., Denicola A. (2013). Evaluating the antioxidant capacity of natural products: A review on chemical and cellular-based assays. Anal. Chim. Acta.

[13] Soto-Vaca A., Gutierrez A., Losso J.N., Xu Z., Finley J.W. (2012). Evolution of phenolic compounds from color and flavor problems to health benefits. J. Agric. Food Chem..

[14] Frijhoff J., Winyard P.G., Zarkovic N., Davies S.S., Stocker R., Cheng D., Knight A.R., Taylor E.L., Oettrich J., Ruskovska T. (2015). Clinical relevance of biomarkers of oxidative stress. Antioxid. Redox Signal..

[15] Donno D., Beccaro G.L., Mellano M.G., Cerutti A.K., Bounous G. (2015). Goji berry fruit (*Lycium* spp.): Antioxidant compound fingerprint and bioactivity evaluation. J. Funct. Foods.

[16] Ceylan R., Katanić J., Zengin G., Matić S., Aktumsek A., Boroja T., Stanić S., Mihailović V., Guler G.O., Boga M. (2016). Chemical and biological fingerprints of two *Fabaceae* species (*Cytisopsis dorycniifolia* and *Ebenus hirsuta*): Are they novel sources of natural agents for pharmaceutical and food formulations?. Ind. Crop. Prod..

[17] Ertaş A., Boga M., Yılmaz M.A., Yeşil Y., Haşimi N., Kaya M.S.E., Temel H., Kolak U. (2014). Chemical compositions by using LC-MS/MS and GC-MS and biological activities of *Sedum sediforme* (Jacq.) Pau. J. Agric. Food Chem..

[18] Eyduran S.P., Ercisli S., Akin M., Beyhan O., Gecer M.K., Eyduran E., Erturk Y.E. (2015). Organic acids, sugars, vitamin c, antioxidant capacity and phenolic compounds in fruits of white (*Morus alba* L.) and black (*Morus nigra* L.) mulberry genotypes. J. Appl. Bot. Food Qual..

[19] Liu R.H. (2003). Health benefits of fruit and vegetables are from additive and synergistic combinations of phytochemicals. Ame. J. Clin. Nutr..

[20] Shahidi F., Ambigaipalan P. (2015). Phenolics and polyphenolics in foods, beverages and spices: Antioxidant activity and health effects—A review. J. Funct. Foods.

[21] Wolfram S., Raederstorff D., Preller M., Wang Y., Teixeira S.R., Riegger C., Weber P. (2006). Epigallocatechin gallate supplementation alleviates diabetes in rodents. J. Nutr..

[22] Škerget M., Kotnik P., Hadolin M., Hraš A.R., Simonič M., Knez Ž. (2005). Phenols, proanthocyanidins, flavones and flavonols in some plant materials and their antioxidant activities. Food Chem..

[23] Wojdyło A., Oszmiański J., Bielicki P. (2013). Polyphenolic composition, antioxidant activity, and polyphenol oxidase (ppo) activity of quince (*Cydonia oblonga* Miller) varieties. J. Agric. Food Chem..

[24] Sánchez-Salcedo E.M., Mena P., García-Viguera C., Martínez J.J., Hernández F. (2015). Phytochemical evaluation of white (*Morus alba* L.) and black (*Morus nigra* L.) mulberry fruits, a starting point for the assessment of their beneficial properties. J. Funct. Foods.

[25] Ştefănuţ M.N., Căta A., Pop R., Moşoarcă C., Zamfir A.D. (2011). Anthocyanins HPLC-DAD and MS characterization, total phenolics, and antioxidant activity of some berries extracts. Anal. Lett..

[26] Landete J.M. (2011). Ellagitannins, ellagic acid and their derived metabolites: A review about source, metabolism, functions and health. Food Res. Int..

[27] Ammar I., Ennouri M., Bouaziz M., Ben Amira A., Attia H. (2015). Phenolic profiles, phytchemicals and mineral content of decoction and infusion of *Opuntia ficus-indica* flowers. Plant Food Hum. Nutr..

[28] Seeram N.P. (2008). Berry fruits: Compositional elements, biochemical activities, and the impact of their intake on human health, performance, and disease. J. Agric. Food Chem..

[29] Papaefthimiou D., Papanikolaou A., Falara V., Givanoudi S., Kostas S., Kanellis A.K. (2014). Genus *Cistus*: A model for exploring labdane-type diterpenes’ biosynthesis and a natural source of high value products with biological, aromatic, and pharmacological properties. Front. Chem..

[30] Trombetta D., Castelli F., Sarpietro M.G., Venuti V., Cristani M., Daniele C., Saija A., Mazzanti G., Bisignano G. (2005). Mechanisms of antibacterial action of three monoterpenes. Antimicrob. Agents Chemother..

[31] Crowell P.L. (1999). Prevention and therapy of cancer by dietary monoterpenes. J. Nutr..

[32] Cazares-Franco M.C., Ramirez-Chimal C., Herrera-Hernandez M.G., Nunez-Colin C.A., Hernandez-Martinez M.A., Guzman-Maldonado S.H. (2014). Physicochemical, nutritional and health-related component characterization of the underutilized mexican serviceberry fruit *Malacomeles denticulata* (Kunth) G. N. Jones. Fruits.

[33] Monsen E.R. (2000). Dietary reference intakes for the antioxidant nutrients: Vitamin C, vitamin E, selenium, and carotenoids. J. Am. Diet. Assoc..

[34] Gonzalez-Molina E., Moreno D.A., Garcia-Viguera C. (2008). Genotype and harvest time influence the phytochemical quality of fino lemon juice (*Citrus limon* (L.) Burm. F.) for industrial use. J. Agric. Food Chem..

[35] Sánchez-Rangel J.C., Benavides J., Heredia J.B., Cisneros-Zevallos L., Jacobo-Velázquez D.A. (2013). The Folin–Ciocalteu assay revisited: Improvement of its specificity for total phenolic content determination. Anal. Methods.

[36] Lee J., Durst R.W., Wrolstad R.E. (2005). Determination of total monomeric anthocyanin pigment content of fruit juices, beverages, natural colorants, and wines by the ph differential method: Collaborative study. J. AOAC Int..

[37] Benzie I.F., Strain J.J. (1999). Ferric reducing/antioxidant power assay: Direct measure of total antioxidant activity of biological fluids and modified version for simultaneous measurement of total antioxidant power and ascorbic acid concentration. Meth. Enzym..

[38] Mok D.K.W., Chau F.T. (2006). Chemical information of Chinese medicines: A challenge to chemist. Chemom. Intell. Lab. Syst..

